# The Growing Problem of Multidrug-Resistant Tuberculosis in North Korea

**DOI:** 10.1371/journal.pmed.1001486

**Published:** 2013-07-30

**Authors:** Kwonjune J. Seung, Stephen W. Linton

**Affiliations:** 1 Eugene Bell Foundation, Washington, D.C., United States of America; 2 Partners In Health, Boston, Massachusetts, United States of America; 3 Brigham and Women's Hospital, Division of Global Health Equity, Boston, Massachusetts, United States of America; 4 Harvard Medical School, Boston, Massachusetts, United States of America

## Abstract

Kwonjune Seung and Stephen Linton from the non-governmental organization EugeneBell discuss the worryingly high levels of multidrug-resistant tuberculosis they have observed in North Korea's tuberculosis sanatoria.

*Please see later in the article for the Editors' Summary*

**Summary Points**

- In the absence of surveillance data, international aid to the North Korean national tuberculosis (TB) program has largely ignored the possibility that drug-resistant TB strains are widespread, as is the case in Russia and China.
- EugeneBell, a non-governmental organization (NGO) that works with North Korean TB patients, believes that spread of multidrug-resistant (MDR) TB in North Korea is much more advanced than previously assumed, based on anecdotal reports from North Korean clinicians and data collected from TB sanatoria.
- Until effective treatment can be made available to them, MDR TB patients will continue to be a source of transmission to families, neighbors, health providers, and other patients.
- Rapid scale-up of MDR TB treatment is possible in North Korea, but only if the international community—including donors, NGOs and governments—takes the threat of MDR TB seriously.

## Tuberculosis in North Korea

Tuberculosis (TB) has long been one of the most serious public health problems in North Korea (Democratic People's Republic of Korea). The estimated TB incidence of 345/100,000 population is higher than in some countries with generalized HIV/AIDS epidemics [1]. HIV is thought to be almost nonexistent in North Korea, but chronic malnutrition, an important risk factor for TB, has become a fact of life for much of the population since the 1990s [2]. The North Korean health care system has been devastated by the economic problems of the past two decades, making it difficult to respond to poverty- and nutrition-related diseases such as TB [3]. In 1998, the Ministry of Public Health (MOPH) adopted DOTS (Directly Observed Treatment, Short-course), the World Health Organization (WHO)-recommended approach for TB control in resource-limited settings. The major components of DOTS—sputum smear microscopy for diagnosis, and standardized regimens of quality-assured drugs for treatment—had not been as strongly emphasized in North Korea previously. In 2003, North Korea began procurement of TB drugs from the Global Drug Facility (GDF), a WHO-led initiative that has supplied high-quality drugs to over 90 countries since its inception in 2001 [4]. In 2010, the Global Fund to Fight AIDS, TB and Malaria (GFATM) began a five-year, US$41.1M project to continue and expand these initiatives [5]. UNICEF, the principal recipient, and WHO, the technical lead, are responsible for implementation of this project.

Little of this international aid has included treatment of drug-resistant TB. For example, in 2011, North Korea reported 85,564 new cases of TB, the vast majority of whom would have received the GDF standard treatment kit for new patients (category I), a red/white cardboard box containing two months of isoniazid, rifampicin, ethambutol, and pyrazinamide, followed by four months of isoniazid and rifampicin. There were also 13,507 patients with a past history of TB treatment who would have received the GDF standard retreatment kit (category II): two months of streptomycin injections plus isoniazid, rifampicin, ethambutol, and pyrazinamide, followed by one month of isoniazid, rifampicin, ethambutol, and pyrazinamide, followed by five months of isoniazid, rifampicin, and ethambutol. These two regimens have been shown to be highly effective for drug-susceptible TB, but are significantly less effective for drug-resistant TB [6].

There has never been any clear scientific evidence that drug-resistant TB is a serious problem in North Korea. North Korea does not have any system for drug resistance surveillance, nor has it ever performed a national or subnational drug resistance survey [7]. The GFATM focus on treatment for drug-susceptible TB would make programmatic sense if the overall proportion of North Korean TB patients with drug resistance were low. In such settings, improving the diagnosis and treatment of drug-susceptible TB can prevent the creation of drug resistance. It is much more difficult to diagnose and treat drug-resistant TB, particularly multidrug-resistant (MDR) TB, defined as resistance to isoniazid and rifampicin, the two strongest TB drugs and the backbone of standard DOTS regimens. MDR TB requires 18–24 months of treatment with expensive and poorly tolerated second-line TB drugs.

In the absence of drug resistance surveillance data, the successful implementation of DOTS has suggested that drug resistance should not be a problem in North Korea. According to WHO, the outcomes for new smear-positive patients receiving category I in 2010 were 86% cured, 4% completed, 3% died, 4% failed, 2% defaulted (1% not evaluated). Outcomes of retreatment patients (category II) were 76% cured, 8% completed, 4% died, 8% failed, 3% defaulted (2% not evaluated) [1]. North Korea is one of the few countries in the world that have attained the WHO target of 90% success (cured plus completed) for new patients, a monumental achievement given the challenges faced by the MOPH. WHO programmatic indicators, however, can exaggerate cure rates because they rely on the relatively insensitive tool of smear microscopy. A patient who is sputum smear-negative after completing a standard DOTS regimen is considered to be a cure, even if there is a small amount of viable bacteria in the sputum that would be detectable by a more sensitive test like culture. This is rarely an issue in settings where almost all TB is drug-susceptible, but in settings where drug-resistant TB is more common, such as Siberia, WHO indicators can overestimate the efficacy of standard DOTS regimens [8],[9].

The experience of countries such as Russia, Azerbaijan, and Uzbekistan, which like North Korea experienced economic hardship after the collapse of the Soviet Union, suggests that drug-resistant TB may already be a serious problem in North Korea. In these countries, the breakdown of health systems and social services was followed rapidly by the spread of MDR strains. These countries continue to struggle with some of the highest rates of MDR TB in the world [1].

## EugeneBell's Experience in North Korea

EugeneBell is a name shared by two non-governmental organizations (NGO), one American, the Eugene Bell Foundation, and one South Korean (Republic of Korea), EugeneBell Korea, which have supported North Korean TB sanatoria throughout the country for over a decade with medicines, equipment, and consumables [10]. On the basis of our experience of working with North Korean TB patients, anecdotal reports from North Korean clinicians, and the original data that is presented here, we believe that the spread of MDR TB is much more advanced than previously assumed.

North Korea has a network of TB sanatoria similar to those that were common in the United States and Europe in the pre-antibiotic era. Dozens of sanatoria exist throughout the country, generally in rural areas, each housing tens to hundreds of patients in cramped conditions. In the past, sanatoria may have admitted only those TB patients who needed injections or who were too sick to take care of themselves at home. But after the widespread implementation of DOTS, many North Korean sanatorium clinicians have reported to us that an increasing number of patients do not seem to be responding to treatment with standard DOTS regimens.

In 2007, after extensive discussions between the MOPH and EugeneBell, six sanatoria in North/South Pyongan provinces and Pyongyang and Nampo cities were designated by the MOPH as MDR TB treatment centers. The combined patient census of these six sanatoria is approximately 800. As part of the new treatment program, we began to develop a system for testing patients for drug resistance. Sputum samples were collected during EugeneBell delegation visits scheduled at six-month intervals. Before each visit, North Korean clinicians pre-selected patients with a history of treatment failure with standard DOTS regimens. Most patients had previously received two or more courses of treatment with DOTS regimens, including category II. Sputum specimens were transported to the Korean Institute of Tuberculosis, a South Korean laboratory that is part of the supranational reference laboratory network that monitors drug-resistant TB globally [1]. Culture and susceptibility testing were performed on Löwenstein-Jensen media to first- and second-line drugs. Pyrazinamide susceptibility was determined using the pyrazinamidase test.

**Figure 1:**
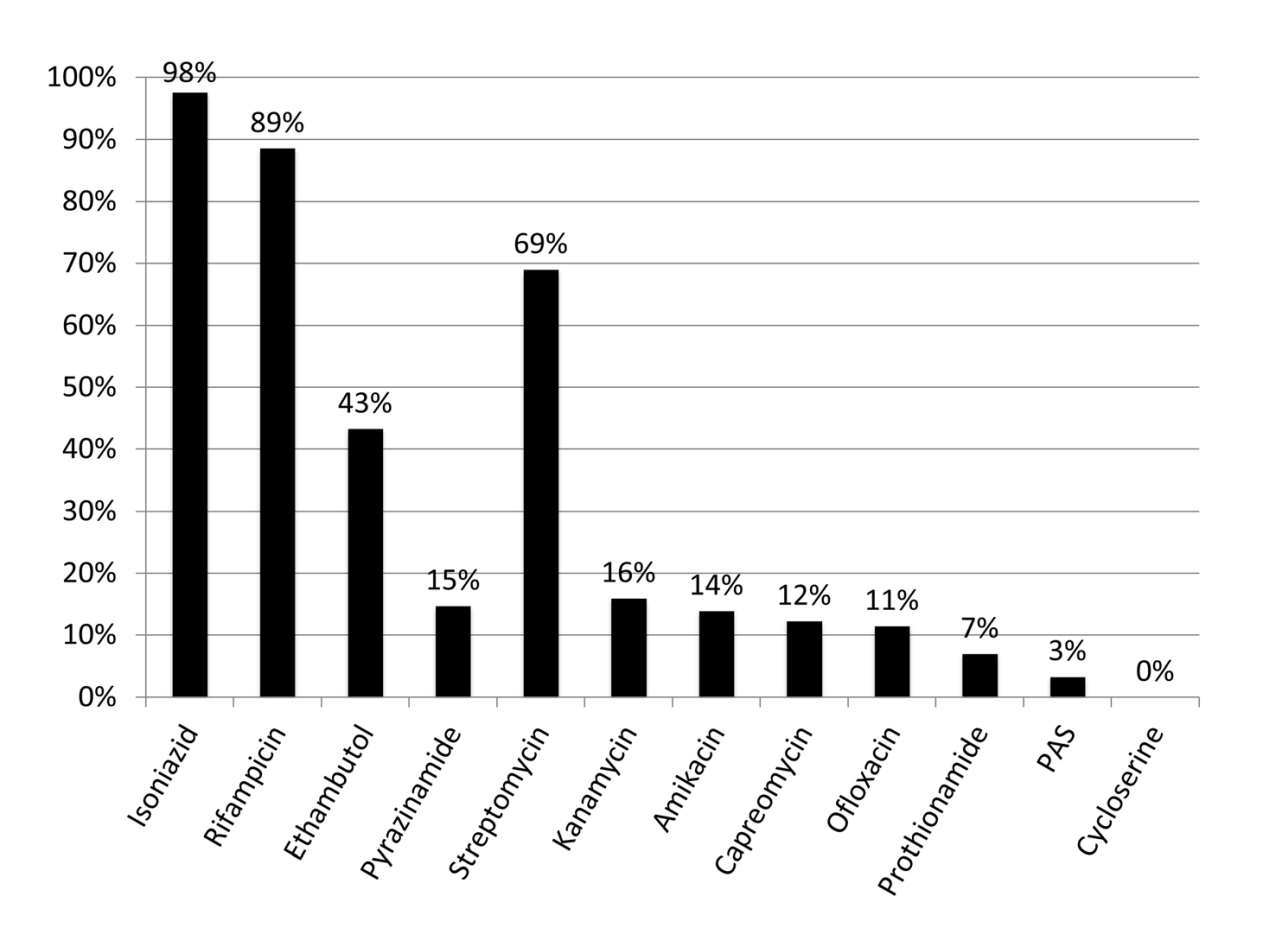
Drug resistance in North Korean sanatorium patients (*n* = 245). The TB Ag MPT64 Rapid kit (SD Bioline), an immunochromatographic test using mouse monoclonal antibodies to detect the MPT64 protein, was used on positive cultures for identification of Mycobacterium tuberculosis complex. Critical concentrations were as follows (mcg/ml): isoniazid 0.2, rifampicin 40.0, ethambutol 2.0, streptomycin 10.0, kanamycin 40.0, amikacin 40.0, capreomycin 40.0, ofloxacin 2.0, prothionamide 40.0, cycloserine 30.0, para-aminosalicylic acid (PAS) 1.0. Pyrazinamide susceptibility was determined using the pyrazinamidase test.

Figure 1 shows the results of drug susceptibility testing of all 245 culture-positive candidates for MDR TB treatment that were presented to EugeneBell during three visits spanning April 2010 to April 2011. Of these, 175 (67%) were men. The median age was 40 (range 32–46). Two-hundred and thirteen (87%) patients were diagnosed with MDR TB (resistance to isoniazid and rifampicin). Thirty-five (14.3%) were diagnosed with MDR plus resistance to a second-line injectable (kanamycin or capreomycin), and 21 (8.6%) were diagnosed with MDR plus resistance to ofloxacin. Six patients (2.4%) were diagnosed with extensively drug-resistant (XDR) TB (MDR plus resistance to a second-line injectable and ofloxacin). The most common MDR patterns are shown in Table 1.

**Table 1:** Common MDR patterns in North Korean sanatorium patients (*n* = 245).

Resistance pattern	Percent (number)
MDR without second-line drug resistance	62% (152)
Isoniazid and rifampicin	12% (30)
Isoniazid, rifampicin, and ethambutol	3% (8)
Isoniazid, rifampicin, and streptomycin	21% (52)
Isoniazid, rifampicin, ethambutol, and streptomycin	18% (43)
Isoniazid, rifampicin, ethambutol, streptomycin, and pyrazinamide	4% (10)
MDR with second-line drug resistance	25% (61)
MDR with second-line injectable resistance	14% (35)
MDR with ofloxacin resistance	9% (21)
XDR	2% (6)

## How Much MDR TB Is There in North Korea?

These resistance data are from a convenience sample of high-risk patients enrolling in a clinical treatment program, and cannot be assumed to be representative of the general TB population. But in the absence of national surveillance data, these data are disturbing. The patient data reported here likely represent a large pool of chronic MDR TB patients that has been growing for years due to the absence of effective treatment.

Strains resistant to second-line TB drugs, including some XDR strains, were present in the patients tested by EugeneBell. Since all patients were tested prior to enrolment in the EugeneBell treatment program, this suggests that some patients had taken second-line TB drugs previously, or had been infected by someone who had. Without access to effective treatment within formal health systems, North Korean MDR TB patients, like MDR TB patients in other countries, will turn to informal systems to obtain second-line TB drugs. Such drugs will inevitably be inadequate in quantity or quality, and likely to result in even more highly resistant strains that can be transmitted to family and neighbors.

Drug resistance surveillance studies are urgently needed to estimate the prevalence of MDR TB in the general TB patient population. WHO estimates there are approximately 3,500 MDR TB cases per year in DPRK, on par with relatively low MDR prevalence countries in the region like India (2.1% of new cases and 15% of retreatment cases). If North Korea's MDR rates were similar to China (5.7% of new cases and 26% of retreatment cases), there would be more than 8,000 MDR TB cases per year. In a worst case scenario, if North Korea's MDR rates were similar to Russia (20% of new cases and 46% of retreatment cases), there would be over 23,000 MDR TB cases per year [1].

## Rapid Scale-up of MDR-TB Treatment Is Urgently Needed in North Korea

At the current time, the most urgent problem in North Korea is the lack of access to the second-line TB drugs needed to treat MDR TB. MDR TB treatment regimens are hundreds of times more expensive than the first-line DOTS regimens used to treat drug-susceptible TB. Many resource-limited countries are only able to procure second-line TB drugs through international funding mechanisms such as the GFATM. In North Korea, however, the GFATM workplan includes procurement of hundreds of thousands of GDF category I and II treatment kits, but only 500 MDR TB treatment courses during the first four years of the project [11].

The lack of laboratory capacity for diagnosis of MDR TB is equally worrisome. The GFATM project focuses mostly on strengthening the national smear microscopy network. North Korea still does not have a TB laboratory accredited to perform drug susceptibility testing, though an international initiative to create such a laboratory in Pyongyang has been underway since 2009 [12],[13]. Ultimately, much more laboratory capacity will be needed to screen over 15,000 retreatment patients annually for MDR TB and to monitor the thousands of MDR TB patients that need treatment.

Since second-line TB drugs have been largely unavailable outside the few MDR TB treatment sites supported by EugeneBell, most North Korean clinicians have no other option except to prescribe repeated courses of first-line TB drugs to patients suspected of having MDR TB. MDR TB patients who are treated empirically with the standard DOTS regimens may become transiently sputum smear-negative and deemed “cured” [14]. When they become smear-positive again, these patients are categorized as “relapses” according to national TB treatment guidelines and treated with the standard DOTS regimen for retreatment patients. Before being diagnosed with MDR TB by EugeneBell, most of the patients included in this report had already received multiple courses of treatment with standard DOTS regimens of first-line drugs that were mostly ineffective. This dangerous practice is likely to be a major factor in the creation of MDR strains in North Korea via the amplifier effect of short-course chemotherapy [15],[16].

Given the urgent need for MDR TB treatment, EugeneBell has stepped into the aid vacuum and has provided support for the North Korean MDR TB treatment program, including second-line TB drugs, laboratory equipment and consumables, and clinical training for North Korean doctors and nurses, who have generally shown exceptional dedication and motivation to treat these most difficult of TB patients. Our budget is small compared to many of the international organizations already supporting the North Korean TB program, but it is almost completely funded by individuals and churches that are drawn to our objective of complete aid transparency. In this manner, EugeneBell acts as a channel for private citizens in North Korea, South Korea, and the US to cooperate and address a serious public health problem.

We have uncovered evidence of a growing MDR TB epidemic, and have demonstrated that North Korean doctors and nurses are ready to treat MDR TB if given the tools to do so. National scale-up of MDR TB diagnosis and treatment, however, will require significantly more resources. The progress of this project has shown that MDR TB treatment scale-up can and should be a major component of North/South cooperation. A comprehensive response to MDR TB would include nutritional supplementation for TB patients, improving housing for sanatorium patients, increasing laboratory capacity to do culture and drug susceptibility testing, procurement of drugs for MDR/XDR TB, and building surgical capacity to do pulmonary resection. Rapid scale-up of MDR TB treatment is possible in North Korea, but only if the international community—including donors, NGOs and governments—takes the threat of MDR TB seriously.

## Supporting Information

Text S1 **Korean translation of the article by EugeneBell.**
(DOCX)
