# Supplementary material for: The Growing Problem of Multidrug-Resistant Tuberculosis in North Korea
Source: PLoS Med. 2013 Jul 30;10(7):e1001486. doi: 10.1371/journal.pmed.1001486 (PMC3727921; doi:10.1371/journal.pmed.1001486)
Supplement: Text S1 — Korean translation of the article by EugeneBell. (DOCX) [file pmed.1001486.s001.docx]

**증가하는 북한 다제내성결핵의 위험성**

승권준Kwonjune J. Seung MD^1,2,3,4^ (교신저자), 인세반Stephen W. Linton, PhD^1^

^1^EugeneBell USA, 207 C Street S.E., Washington DC, USA

^2^Partners In Health, 888 Commonwealth Ave., Boston USA

^3^Brigham and Women's Hospital, Division of Global Health Equity, 75 Francis St., Boston USA

^3^Harvard Medical School, 250 Longwood Ave., Boston, USA

**내용 요약**

- 공식적인 통계 자료의 부재로 인해, 북한의 국가결핵대책계획을 지원하기 위한 국제 사회의 지원은 러시아와 중국의 경우와 마찬가지로 다제내성결핵 확산 가능성을 크게 간과한 측면이 있다.
- 북한 결핵 환자를 지원하는 비정부기구인 유진벨 재단은 북한 의료진의 비공식 보고서와 북한 요양소에서 수집된 자료를 근거로 북한 내 다제내성결핵 확산 정도가 예상했던 수준보다 더욱 심각한 것으로 판단하고 있다.
- 효과적인 다제내성결핵 치료 계획이 도입되기 전까지 다제내성결핵 환자들은 그들의 가족, 이웃, 의료진과 다른 환자에게 계속 다제내성결핵을 감염시킬 것이다.
- 북한 다제내성결핵 치료의 신속한 확장은 오직 국제사회 (후원자, 비정부기구와 정부를 포함하여)가 다제내성결핵의 위험성을 심각하게 받아들이는 경우에만 가능할 것이다.

***북한 결핵 현황***

결핵은 오랜 기간 북한에서 가장 심각한 보건의료 문제 중 하나로 다루어져 왔다. 북한의 결핵 발생률 10만명당 345명으로, 에이즈(HIV/AIDS)가 만연된 일부 국가보다도 높은 수준이다.^[[1]](#endnote-1)^ 북한은 에이즈(HIV/AIDS)가 거의 발생하지 않는 것으로 알려져 있다. 그러나 1990년대 고난의 행군 이후 많은 북한주민이 결핵의 가장 중요한 원인인 만성 영양실조에 걸리게 되었다.^[[2]](#endnote-2)^ 지난 20년간의 경제난으로 북한의 공중 보건의료 체계가 약화되어, 결핵과 같이 빈곤, 영양과 관련된 질병에 대처하는 것을 더욱 어렵게 하고 있다.^[[3]](#endnote-3)^ 1998년 북한은 DOTS (Directly Observed Treatment, Short-course)를 채택했는데, 세계보건기구(WHO)가 자원이 제한된 지역의 결핵퇴치전략으로 권장한 것이었다. DOTS의 가장 중요한 요소는 결핵 진단을 위한 객담 도말 검사, 품질이 검증된 약품으로 이루어진 표준처방이다. 2003년에 북한은 국제의약시설(Global Drug Facility, GDF)로 부터 결핵약 지원을 받기 시작했다. 국제의약시설은 세계보건기구 산하 기관으로, 2001년에 설립된 이래 90개국에 뛰어난 품질의 결핵약을 공급해오고 있다.^[[4]](#endnote-4)^ 2010년에는 에이즈, 결핵, 말라리아 퇴치 세계기금(the Global Fund to Fight AIDS, TB and Malaria (GFATM))이 이 계획을 지속 및 확장 하기 위하여 5년간 4,110만 달러를 투입하는 계획을 시작했다.^[[5]](#endnote-5)^ 유니세프(UNICEF)가 주체가 되고 세계보건기구가 의학부분을 담당하여 계획을 실행하고 있다.

이 같은 국제사회 원조의 극히 일부분만이 다제내성결핵 치료에 투입된다. 예를 들면, 2011년에 북한은 85,564명의 결핵 신환자수를 보고했는데, 이들 대부분은 국제의약시설이 공급한 표준 치료(1부류, Category I)를 받았다. 빨간색과 흰색으로 되어있는 이 치료 키트에는 2개월 동안 복용할 수 있는 아이나(Isoniazid), 리팜피신(Rifampicin), 에탐부톨(Ethambutol), 피라진아미드(Pyrazinamide)와 이후 4개월을 더 복용할 수 있는 아이나(Isoniazid)와 리팜피신(Rifampicin)이 들어있다. 이전에 결핵 치료를 받은 적이 있는 13,507명의 환자(재치료 환자)는 국제의약시설이 공급한 표준 재치료(2부류, Category II)를 제공받는다. 이 표준 재치료 키트에는 2개월 동안 환자를 치료할 수 있는 아이나(Isoniazid)를 포함하여 스트렙토마이신(Streptomycin) 주사약, 리팜피신(Rifampicin), 에탐부톨(Ethambutol), 피라진아미드(Pyrazinamide)와 이후 1개월을 더 복용할 수 있는 아이나(Isoniazid), 리팜피신(Rifampicin), 에탐부톨(Ethambutol), 피라진아미드(Pyrazinamide)가 제공되며, 그리고 이후 5개월을 더 복용할 수 있는 아이나(Isoniazid), 리팜피신(Rifampicin), 에탐부톨(Ethambutol)이 들어 있다. 이 2가지의 표준 처방은 일반적으로 약제내성이 없는 결핵에는 큰 효과를 보였으나, 약제내성결핵에는 그리 효과적이지 못했다.^[[6]](#endnote-6)^

지금까지 북한에서는 약제내성결핵이 심각한 문제라는 것을 증명할 과학적 근거가 충분하지 않았다. 북한은 약제내성 감시체계가 전혀 없었으며, 국가나 지역차원의 약제내성률 조사를 시행한 적도 없었다.^[[7]](#endnote-7)^ 북한 전체 결핵환자 중 약제내성 환자의 비율이 낮다면 약제내성이 없는 일반결핵 치료에 집중된 세계기금의 계획은 상당한 효과를 거둘 수 있었을 것이다. 약제내성이 없는 일반결핵이 대부분인 경우에는, 일반결핵을 대상으로 한 진단과 치료규모의 확대만으로 약제내성의 발생을 막을 수 있다. 그러나 일반결핵이 아닌 약제내성결핵의 진단과 치료는 훨씬 어렵고, 그 중에서도 일반결핵치료에 가장 큰 역할을 하는 아이나(Isoniazid)와 리팜피신(Rifampicin)에 내성을 갖게 된 다제내성결핵 (multidrug-resistant TB, MDR TB)을 진단하고 치료하는 것은 더욱 어려운 일이다. 다제내성결핵은 일반 결핵약에 비해 비싸고 부작용 역시 심각한 결핵 2차 약제를 사용하여 18개월에서 24개월 동안 치료해야 한다.

약제내성에 대한 통계자료가 존재하지 않았으므로, 지금까지는 DOTS 요법을 성공적으로 시행하기만 하면 북한에서 약제내성이 큰 문제가 되지 않을 것이라고 추측해 왔다. 세계보건기구에 따르면 2010년 북한에서 객담 도말검사 양성인 1부류 치료를 받은 신환자의 치료 결과는 다음과 같다. 86% 완치, 4% 완료, 3% 사망, 4% 실패, 2% 중단(1% 미확인). 2부류 치료를 받은 재치료 환자의 치료결과는 76% 완치, 8% 완료, 4% 사망, 8% 실패, 3% 중단(2% 미확인)이다. 이 결과로만 보면 북한은 세계보건기구가 목표로 하고 있는 신환자 90% 치료성공(완치와 완료)을 달성한 세계 몇 안 되는 국가 중에 포함된다. 이는 북한 보건성이 당면한 많은 어려움을 고려할 때 인상적인 성과이지만, 한편으로는 세계보건기구의 완치율이 과장된 것으로 보이기도 한다. 왜냐하면 도말 검사라는 민감도가 떨어지는 검사방법에 의존한 지표이기 때문이다. 표준 DOTS 치료과정을 모두 마친 후 객담 도말검사 결과에서 음성으로 나타난 환자는 완치 판정을 받게 된다. 그러나 도말검사 음성이라도 배양검사에서는 양성을 보일 수 있다. 배양검사의 민감도가 더 높으므로 미량의 결핵균도 발견할 수 있기 때문이다. 약제내성이 없는 일반 결핵이 대부분인 경우에는 이것이 큰 문제가 되지 않지만, 시베리아와 같이 약제내성결핵이 쉽사리 발견되는 경우에는, 세계보건기구 기준 지표가 표준 DOTS 치료 효과를 과대평가했을 가능성도 있다.^[[8]](#endnote-8),^^[[9]](#endnote-9)^

북한과 비슷하게 소비에트 연방 해체 이후 심각한 경제난을 겪은 구 소련, 아제르바이잔, 우즈베키스탄의 사례를 보면 이미 북한의 다제내성결핵이 심각한 수준임을 추정할 수 있다. 이러한 나라에서 공중 보건의료 체계와 사회복지제도의 붕괴는 다제내성결핵의 빠른 확산을 가져왔다. 이 국가들은 현재도 세계에서 가장 높은 결핵 다제내성율과 힘겹게 싸우고 있는 중이다.^[[10]](#footnote-1)^

***유진벨의 북한 결핵 치료사업 경험***

유진벨 재단은 미국과 한국에 근거를 두고 활동하고 있으며 10년 이상 의약품, 진단장비, 의료 소모품을 북한의 결핵요양소에 지원한 비정부기구다.^[[11]](#endnote-10)^ 북한 결핵환자를 치료해온 유진벨의 경험과 북한 의료진이 제출한 비공식 보고서, 현재 북한 의료자료 등에 기초하여 보면, 다제내성결핵이 광범위하게 확산되어 있는 것으로 보여진다.

북한은 미국과 유럽이 항생제가 없던 시절에 운영했던 것과 비슷한 수준의 결핵요양소를 전국적으로 운영하고 있다. 수십 개의 요양소가 북한 전역에, 특히 시외 지역에 퍼져 있으며 각 요양소에는 수십에서 수백 명의 환자들이 비좁은 요양소에서 입원 치료를 받고 있다. 과거에는 요양소에서 주사 치료가 필요한 결핵환자나 중환자여서 집에서 치료를 하기 어려운 환자만 수용하여 치료하였다. 그런데 DOTS 요법이 결핵 치료에 광범위하게 사용된 이후에는 북한 요양소 의료진들은 점점 더 많은 수의 환자가 표준 DOTS 처방의 치료에 반응하지 않는 다는 것을 발견하였고, 유진벨에도 이 부분에 대해 알려 왔다.

2007년에 유진벨과 북한 보건성은 이 문제에 대해 진지하게 고민하였고, 그 결과로 평안 남∙북도와 평양시, 남포시의 6개 결핵요양소를 다제내성결핵 센터로 지정하기로 했다. 6개 결핵요양소의 환자는 모두 800여명이었다. 새로운 결핵치료 사업을 시작하면서, 유진벨은 환자들의 약제내성을 검사할 수 있는 체계를 갖추기 시작했다. 유진벨 대표단은 6개월에 1번씩 북한을 방문했고 이때마다 환자의 객담을 채취했다. 매번 북한을 방문하기 전에, 북한 현지 의료진들은 표준 DOTS 처방으로 실패한 병력을 가진 환자들을 선별해 두었다. 대부분의 환자들은 2부류 치료를 포함하여 2번 이상의 DOTS 처방으로 치료를 받았던 환자들이었다. 북한 환자의 객담 검체는 초국가결핵참조검사실인 한국의 결핵연구원으로 옮겨져서 검사 된다. 배양검사와 감수성검사는 Löwenstein-Jensen 배지를 이용하여 1차약제와 2차약제에 대해 이루어졌다. 피라진아미드(Pyrazinamide) 감수성은 Pyrazinamidase 검사를 통하여 확인하였다.

그림 1은 2010년 봄부터 2011년 가을까지, 3번의 방문기간 동안 유진벨에 객담을 제출하여 배양검사 양성으로 확인된 245명의 환자후보자들의 감수성 결과를 보여주고 있다. 245명중 175명(67%)은 남성이었고 환자 연령의 중앙값은 40세였다 (나이분포: 32-46세). 213명(87%)의 환자는 다제내성결핵으로 조사되었다. 35명(14.3%)의 환자는 다제내성결핵일 뿐 아니라 2차 주사약제(카나마이신(Kanamycin)이나 카프레오마이신([Capreomycin](http://endic.naver.com/enkrEntry.nhn?entryId=74913f0fb2224cc18928d1f4f6644c91&query=%EC%B9%B4%ED%94%84%EB%A0%88%EC%98%A4%EB%A7%88%EC%9D%B4%EC%8B%A0)))에도 내성이 있는 것으로 확인되었다. 21명(8.6%)은 다제내성결핵이며서 오플록사신(Ofloxacin)에도 내성이 있음을 확인할 수 있었다. 6명의 환자(2.4%)는 광범위내성결핵(XDR TB)으로 드러났다. 광범위내성결핵이란 아이나(Isoniazid)와 리팜피신(Rifampicin)뿐만 아니라 2차 주사약제와 오플록사신(Ofloxacin)에 내성을 가진 결핵이다. 가장 일반적인 다제내성 패턴은 표 1에서 확인할 수 있다.

***북한에 얼마나 많은 다제내성결핵환자가 있는가?***

위에서 사용한 자료는 내성이 위험성 높은 환자가 등록된 치료사업의 표본이기 때문에 일반 결핵 환자의 대표군으로 사용할 수는 없다. 그러나 국가 차원의 공식적 통계자료가 존재하지 않는 것을 감안할 때, 이 자료는 상당히 심각해 보인다. 앞에서 사용한 환자자료를 검토해 보면 대규모의 만성 다제내성결핵 환자군이 존재하는 것으로 추정되며 이는 효과적인 치료가 없었기 때문에 수년 동안 계속해서 증가해 온 것으로 보인다.

유진벨은 환자 등록 전에 모든 환자들에게 객담 검사를 실시했다. 이들 중에는 소수의 광범위내성결핵환자를 포함하여 2차 약제에 내성을 가진 환자들이 발견된다. 이는 환자들이 등록 전에 2차 약제를 이미 복용한 경험이 있다거나 2차 약제를 이미 복용한 환자로부터 감염되었다는 것을 의미한다. 보건의료체계를 통한 효과적인 치료 접근이 어려운 국가에서는 환자들이 2차 약제를 얻기 위해 검증되지 못한 경로를 이용하게 되는데, 북한도 이와 비슷한 경우로 보여진다. 이런 약들은 당연히 약품의 질이나 양 모두에서 불충분하고 더욱 강한 내성결핵균을 가족과 이웃에게 옮기는 결과를 초래하게 된다.

일반 결핵 환자 중에 다제내성결핵이 얼마나 널리 퍼졌는지를 확인 할 수 있는 약제 내성 감시 연구도 시급한 상황이다. 세계보건기구는 북한에서 연간 3500명 정도의 다제내성결핵환자가 발생한다고 추정하고 있다. 이는 상대적으로 다제내성율이 낮은 인도 정도의 수준이다. (신환자의 2.1%와 재치료환자의 15%). 그러나 만약 북한의 다제내성율이 중국과 비슷하다면 연간 8,000명 이상의 다제내성결핵환자가 발생한다는 말이 된다. (신환자의 5.7%와 재치료환자의 26%). 가장 나쁜 시나리오의 경우는 북한의 다제내성율이 러시아 수준인 경우인데, (신환자의 20%와 재치료환자의 46%). 이 경우는 연간 23,000명 이상의 다제내성결핵환자가 발생한다고 예상할 수 있다.

***북한 다제내성결핵 치료사업의 확장이 시급하다***

현재 북한에서 가장 긴급한 문제는 다제내성결핵 치료를 위한 2차 약제에 대한 접근이 어렵다는 점이다. 다제내성결핵 치료에 사용되는 처방은 내성이 없는 일반결핵을 치료하는데 사용되는 DOTS 처방보다 수백 배 비싸다. 자원이 제한된 많은 국가들은 세계기금과 같은 국제 기구를 통해서만 2차 결핵약제를 공급 받을 수 있다. 그런데 북한에서의 세계기금 실행 계획을 살펴보면 일반결핵약제에 대해서는 수십만명분의 1부류와 2부류 (Category I과 Category II)키트를 제공하기로 되어 있지만, 다제내성결핵에 대해서는 4년 동안 단지 500명의 환자를 치료한다는 계획을 가지고 있을 뿐이다.^[[12]](#endnote-11)^

다제내성결핵진단을 위한 실험실의 처리 용량 부족도 역시 우려된다. 세계기금 계획은 주로 국가 수준의 도말검사 실험실망 강화에 초점이 맞춰져 있다. 북한은 2009년부터 국제적인 연계를 통하여 평양에 실험실 설립을 진행하고 있지만 약제 감수성 검사를 할 수 있는 인증 받은 실험실은 여전히 갖추고 있지 못하다.^[[13]](#endnote-12),^^[[14]](#endnote-13)^ 연간 15,000 명의 재치료 환자 중에서 다제내성결핵 여부를 판별하고, 그 중에 다제내성결핵 치료가 필요한 수천명의 환자를 관리하기 위해서는 실험실의 역량강화가 절대적으로 필요하다.

유진벨이 지원하는 일부 다제내성결핵센터를 제외하고는 2차 결핵 약제를 구하는 것이 거의 불가능하다. 따라서 유진벨지원을 받지 못하는 대부분의 북한 의료진이 할 수 있는 것이라고는 다제내성결핵이 의심되는 환자에게도 1차 결핵약제를 계속 처방하는 것 외에는 다른 방법이 없다. 표준 DOTS 처방으로 치료받은 경험이 있는 다제내성결핵환자들은 일시적인 도말 검사 음성을 “완치” 된 것이라고 생각할 수 있다.^[[15]](#endnote-14)^ 다시 도말 검사 양성이 나타나면 이 환자들은 국가 결핵 치료 지도서에 따라 “재발”로 분류되고 재치료 환자를 위한 표준 DOTS 처방으로 치료받게 된다. 유진벨에 등록된 대부분의 환자들은 다제내성결핵으로 진단받기 전에, 거의 아무 효과가 없는 표준 DOTS 처방으로 수 차례 거듭해서 치료를 받은 경험이 있다. 이 위험한 치료방법이 북한에서 내성결핵을 발생시키는 주원인이다(DOTS로 인한 내성결핵 증폭 효과).^[[16]](#endnote-15)^’^[[17]](#endnote-16)^

다제내성결핵 치료를 위한 시급한 도움이 요구되는 상황에서, 유진벨 재단은 국제사회의 원조 공백을 채우고 북한 다제내성결핵 치료사업을 지원했는데, 여기에는 결핵 2차 약제, 실험실 물품, 의료 소모품뿐만 아니라 결핵 치료에 깊이 헌신한 북한 의료진(의사와 간호사)을 교육하는 내용도 포함되어 있다. 유진벨의 예산은 이미 북한에서 결핵 치료 사업을 하고 있는 다른 국제 기구와 비교하면 적은 편이다. 그렇지만 대부분의 예산이 개인과 교회에 의해 모금되고 있고, 이 후원자들은 철저하게 투명한 지원을 목적으로 하는 유진벨의 원칙에 이끌려서 재단을 후원하고 있다. 이 같은 방식으로 유진벨은 심각한 공중보건 문제에 대하여 함께 협력하고 해결할 수 있도록 북한, 남한, 미국을 연결하는 통로의 역할을 하고 있다.

이 글을 통해 우리는 북한 다제내성결핵 발병 증가에 대한 타당한 증거를 제시했고, 북한의 의사와 간호사가 적절한 지원만 제공된다면 다제내성결핵을 치료할 준비가 되어있음을 보여주었다. 그러나 다제내성결핵의 진단과 치료를 전국적인 규모로 확장하기에는 상당히 많은 자원이 요구된다. 이 사업의 진전은 다제내성결핵치료의 확장이 남북 협력의 중요한 요소가 될 수 있으며, 또 되어야만 한다는 것을 보여준다. 다제내성결핵에 대하여 폭넓게 대응하기 위해서는 환자 영양식, 요양소 환자의 주거 개선, 실험실의 배양검사 및 감수성검사 처리용량 증가, 다제내성/광범위내성 결핵을 위한 약 공급, 폐 절제술을 시행 할 수 있는 외과 시설 구축이 필요하다. 북한 다제내성결핵 치료의 신속한 확장은 오직 국제사회 (후원자, 비정부기구와 정부를 포함하여)가 다제내성결핵의 위험성을 심각하게 받아들이는 경우에만 가능할 것이다.

***감사의 말***

유진벨 다제내성결핵사업의 배양검사와 감수성검사를 지원하고 있는 결핵연구원 김희진 원장님과 김창기 부장님의 노고에 깊이 감사 드린다.

## 그림1: 북한 요양소 환자들의 약제내성분포(N=245)


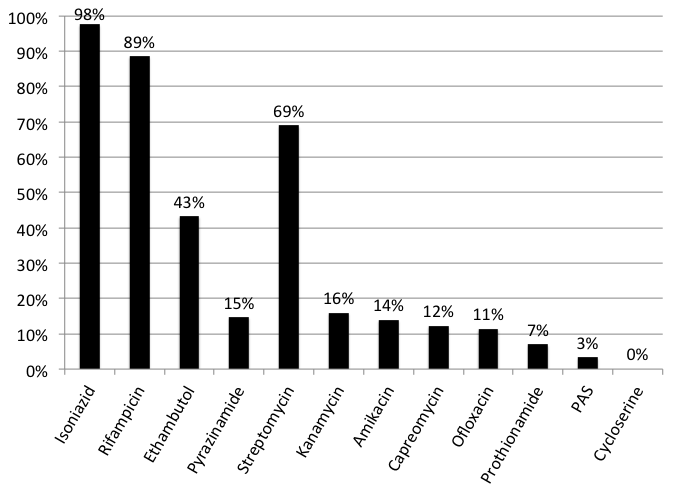


The TB Ag MPT64 Rapid kit (SD Bioline, South Korea), an immunochromatographic test using mouse monoclonal antibodies to detect the MPT64 protein, was used on positive cultures for identification of Mycobacterium tuberculosis complex. Critical concentrations were as follows (mcg/ml): isoniazid 0.2, rifampicin 40.0, ethambutol 2.0, streptomycin 10.0, kanamycin 40.0, amikacin 40.0, capreomycin 40.0, ofloxacin 2.0, prothionamide 40.0, cycloserine 30.0, para-aminosalicylic acid (PAS) 1.0. Pyrazinamide susceptibility was determined using the pyrazinamidase test.

## 표1: 북한 요양소 환자들의 일반적인 약제내성 패턴 (N=245)

| **내성패턴 (Resistance pattern)** | **퍼센트 (환자수)** |
| --- | --- |
| 2차 약제에 내성이 없는 다제내성결핵  (MDR without second-line drug resistance) | 62% (152) |
| 아이나(Isoniazid)와 리팜피신(Rifampicin) | 12% (30) |
| 아이나(Isoniazid), 리팜피신(Rifampicin), 에탐부톨(Ethambutol) | 3% (8) |
| 아이나(Isoniazid), 리팜피신(Rifampicin), 스트렙토마이신(Streptomycin) | 21% (52) |
| 아이나(Isoniazid), 리팜피신(Rifampicin), 에탐부톨(Ethambutol), 스트렙토마이신(Streptomycin) | 18% (43) |
| 아이나(Isoniazid), 리팜피신(Rifampicin), 에탐부톨(Ethambutol), 스트렙토마이신(Streptomycin), 피라진아미드(Pyrazinamide) | 4% (10) |
| 2차 약제에 내성이 있는 다제내성결핵  (MDR with second-line drug resistance) | 25% (61) |
| 다제내성결핵이면서 2차 주사 약제에 내성이 있음 | 14% (35) |
| 다제내성결핵이면서 오플록사신(Ofloxacin)에 내성이 있음 | 9% (21) |
| 광범위내성결핵 (XDR) | 2% (6) |

**참고문헌**

1. 1. World Health Organization (2012). Global tuberculosis control 2012 (WHO/HTM/TB/2012.6). Geneva: World Health Organization. [↑](#endnote-ref-1)
2. . Food and Agriculture Organization/World Food Program (2011). Crop and food security assessment mission to the Democratic People's Republic of Korea, 25 November 2011. Available: http://www.fao.org/docrep/014/al982e/al982e00.htm. Accessed 15 Feb 2013. [↑](#endnote-ref-2)
3. . WHO Regional Office for South-east Asia (2009). WHO Country Cooperation Strategy: Democratic People's Republic of Korea: 2009-2013. Available: <http://www.who.int/countryfocus/cooperation_strategy/ccs_prk_en.pdf>. Accessed 15 Feb 2013. [↑](#endnote-ref-3)
4. . Matiru R, Ryan T (2007). The Global Drug Facility: a unique, holistic and pioneering approach to drug procurement and management. *Bull World Health Organ* 85(5): 348-53. [↑](#endnote-ref-4)
5. . Tuberculosis in DPR Korea. Available: <http://www.dprk.searo.who.int/EN/Section16_55.htm>. Accessed 15 Feb 2013. [↑](#endnote-ref-5)
6. . Lew W, Pai M, Oxlade O, Martin D, Menzies D (2008). Initial drug resistance and tuberculosis treatment outcomes: systematic review and meta-analysis. *Ann Intern Med* 149(2): 123-34. [↑](#endnote-ref-6)
7. . World Health Organization (2010). Multidrug and extensively drug resistant tuberculosis (M/XDR-TB): 2010 global report on surveillance and response (WHO/HTM/TB/2010.3). Geneva: World Health Organization. [↑](#endnote-ref-7)
8. . Farmer P (1999). Managerial successes, clinical failures. *Int J Tuberc Lung Dis* 3(5): 365-7. [↑](#endnote-ref-8)
9. . Migliori GB, Espinal M, Danilova ID, Punga VV, Grzemska M, Raviglione MC (2002). Frequency of recurrence among MDR-TB cases ‘successfully’ treated with standardized short-course chemotherapy. *Int J Tuberc Lung Dis* 6(10): 858-864. [↑](#endnote-ref-9)
10. [↑](#footnote-ref-1)
11. . Eugene Bell Foundation. Available: http://www.eugenebell.org. Accessed 15 Feb 2013. [↑](#endnote-ref-10)
12. . Korea (Democratic Peoples Republic): Strengthening Tuberculosis Control in DPR Korea. Available: <http://portfolio.theglobalfund.org/en/Grant/Index?grantNumber=PRK-810-G02-T>. Accessed 15 Feb 2013. [↑](#endnote-ref-11)
13. . Parry J (2010). North Korea’s fight against tuberculosis gets a boost. *BMJ* 340: c2223. [↑](#endnote-ref-12)
14. . Perry S, Linton H, Schoolnik G (2011). Tuberculosis in North Korea. *Science* 331 (6015): 263. [↑](#endnote-ref-13)
15. . Seung KJ (2010). Does my patient have multidrug-resistant tuberculosis? *Clin Infect Dis* 51(4): 379-80. [↑](#endnote-ref-14)
16. . Seung KJ, Gelmanova IE, Peremitin GG, Golubchikova VT, Pavlova VE, et al (2004). The effect of initial drug resistance on treatment response and acquired drug resistance during standardized short-course chemotherapy for tuberculosis. *Clin Infect Dis* 39(9): 1321-8. [↑](#endnote-ref-15)
17. . Cox HS, Niemann S, Ismailov G, Doshetov D, Orozco JD, et al (2007). Risk of acquired drug resistance during short-course directly observed treatment of tuberculosis in an area with high levels of drug resistance. *Clin Infect Dis* 44(11): 1421-7. [↑](#endnote-ref-16)
